# Case Report: Novel ASAP1::BRAF fusion in a young adult with low-grade temporal lobe glioma

**DOI:** 10.3389/fonc.2026.1763047

**Published:** 2026-01-23

**Authors:** Petroula Gerasimou, Dimitris Vrachnos, Yiannos Kyprianou, Agathi Elpidoforou, Andri Miltiadous, Andri Mitsidou, Katerina Nicolaou, Gabriella Shianiou, Christina Zartila, Christina Ioannou, Andrea Christofi, Efi Georgiou, Chrysanthi Avgousti, Maria Kanezou, Marina Johnson, Varnavas Papanastassiou, Christina Oxinou, Marilena Theodorou, Anna Maria Shiarli, Athos Antoniades, Jianxiang Chi, Paul Costeas

**Affiliations:** 1 Molecular Hematology-Oncology Department, Karaiskakio Foundation, Nicosia, Cyprus; 2 Stremble Ventures Ltd., Limassol, Cyprus; 3 Neurosurgery Department, Aretaieio Hospital, Nicosia, Cyprus; 4 Christina Oxinou Histopathology/Cytology Laboratory, Nicosia, Cyprus; 5 Department of Radiation Oncology & Nuclear Medicine, Bank of Cyprus Oncology Center, Nicosia, Cyprus; 6 Centre for the Study of Haematological and other Malignancies, Strovolos, Cyprus; 7 Karaiskakio Foundation, Nicosia, Cyprus

**Keywords:** novel fusion, BRAF fusion, low-grade glioma, next-generation sequencing, FusionAI

## Abstract

Alterations involving the mitogen-activated protein kinase (MAPK) pathway are central drivers of pediatric and adult low-grade gliomas (LGGs), with BRAF fusions representing a dominant oncogenic mechanism in pilocytic astrocytoma. While KIAA1549::BRAF remains the most prevalent fusion, an expanding repertoire of alternative fusion partners continues to refine the molecular landscape of MAPK-driven gliomas and has important therapeutic implications. Here, we report a previously unrecognized ASAP1::BRAF fusion identified in a young adult with a World Health Organization grade 1 temporal lobe pilocytic astrocytoma, highlighting both its biological plausibility and potential relevance for targeted therapy. A 31-year-old female presented with new-onset seizures and underwent gross total resection of a well-circumscribed, partially cystic left temporal lobe tumor. Histopathological and immunohistochemical findings were consistent with pilocytic astrocytoma, demonstrating low proliferative activity and absence of high-grade features. Comprehensive molecular profiling using RNA-based next-generation sequencing revealed an in-frame ASAP1 exon 29::BRAF exon 9 fusion, preserving the intact C-terminal BRAF kinase domain while eliminating N-terminal regulatory regions. No additional pathogenic variants were detected. To substantiate the structural authenticity of the fusion, deep learning–based breakpoint validation using FusionAI was performed, yielding a high fusion probability score and supporting a bona fide genomic rearrangement rather than an RNA-sequencing artifact. Genomic feature annotation demonstrated enrichment of repetitive elements, regulatory regions, and chromatin accessibility features flanking the breakpoint, consistent with known mechanisms of fusion gene formation. Functionally, the ASAP1::BRAF fusion is predicted to emphasize constitutive MAPK pathway activation via dimer-dependent BRAF signaling, analogous to canonical BRAF fusions and mechanistically distinct from BRAF V600E mutations. Clinically, BRAF fusion–driven tumors are typically resistant to first-generation BRAF inhibitors but may be sensitive to MEK inhibitors or emerging type II RAF inhibitors that effectively target RAF dimers. Although no adjuvant therapy was required following complete resection, documentation of this fusion provides a rational framework for future molecularly guided treatment should disease recurrence occur. This case expands the spectrum of oncogenic BRAF fusion partners in LGG and underscores the importance of integrated RNA-based diagnostics and computational validation in precision neuro-oncology.

## Introduction

BRAF gene alterations play a pivotal role in the development of low-grade gliomas (LGGs), particularly through activation of the mitogen-activated protein kinase (MAPK) pathway. Among the fusion variants, KIAA1549::BRAF is the most common, particularly in pilocytic astrocytomas (1). However, novel BRAF fusion partners continue to emerge, expanding the known landscape of MAPK-driven gliomas.

A comprehensive molecular analysis of >1,000 pediatric LGGs demonstrated that 84% harbored a MAPK-pathway driver alteration, with rearrangement-driven tumors, particularly BRAF fusions, occurring at younger ages, exhibiting a predominance of WHO grade I histology, and showing significantly lower rates of progression and disease-related mortality relative to SNV-driven counterparts (2). More recent multi-institutional profiling of >300 pediatric and adult gliomas further delineated age-dependent differences in BRAF-mutant tumors, identifying a higher prevalence of BRAF V600E and BRAF fusion events in pediatric cases, whereas in adults BRAF V600E status was associated with improved survival and enhanced responsiveness to targeted MAPK-pathway inhibition (3).

Emerging research has identified a growing spectrum of alternative BRAF fusion partners beyond KIAA1549, including *CTTNNBP2, SLC44A1* and FYCO1, which similarly drive oncogenic signaling (4). A single-institutional study by Ali et al. (5) (5) confirmed the presence of various BRAF and non-BRAF MAPK pathway alterations across pediatric and adult gliomas, emphasizing the diagnostic value of RNA-based fusion detection.

Therapeutically, the use of MEK inhibitors like trametinib has shown efficacy in BRAF fusion-positive LGGs, highlighting the clinical relevance of molecular diagnostics in guiding treatment (6). Crotty et al. (7) further argue for the incorporation of molecular-targeted therapy into standard care, particularly for MAPK-driven tumors. Emerging Type II RAF inhibitors such as tovorafenib and belvarafenib have demonstrated activity against dimer-driven BRAF fusion signaling, offering a mechanistically rational therapeutic strategy for MAPK-driven brain tumors.

## Case presentation

**Figure 1:**
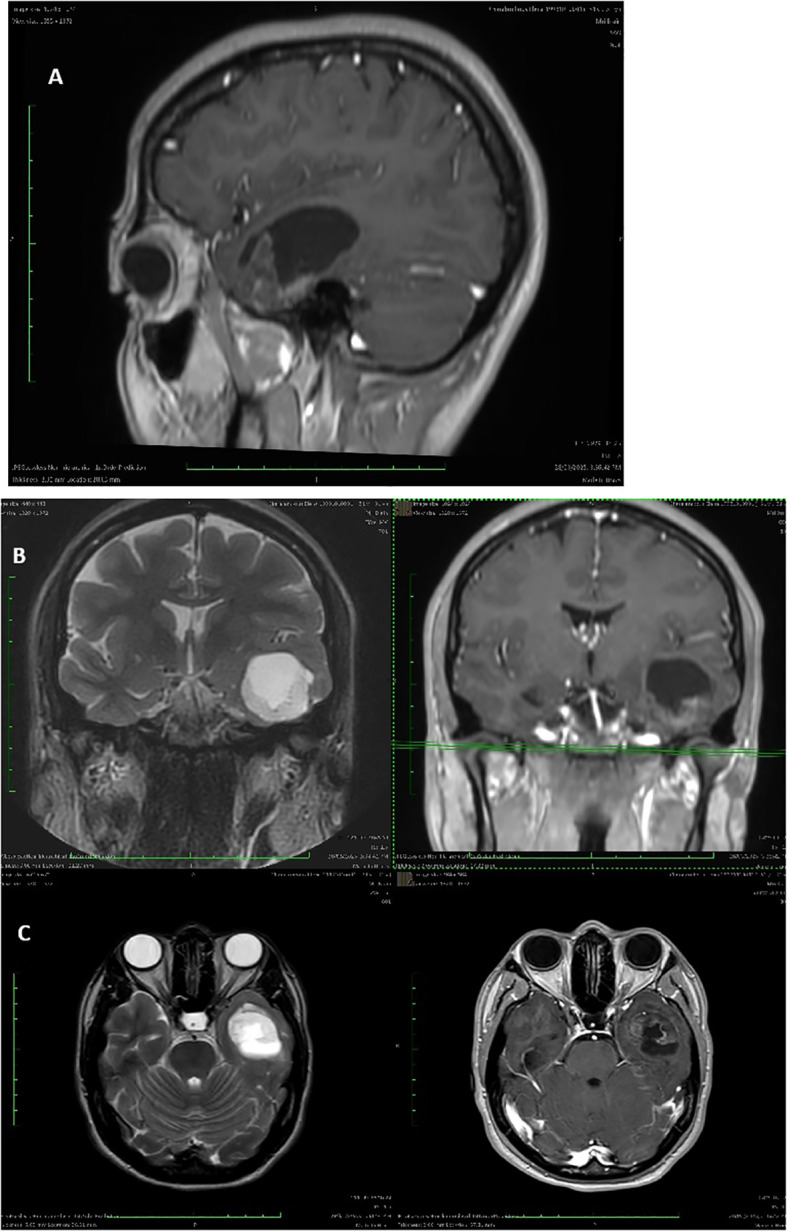
**(A)** Sagittal post-contrast T1-weighted MRI demonstrating a partially cystic lesion localized to the left temporal pole. The lesion shows mild peripheral enhancement and exerts mass effect on the adjacent lateral ventricle, consistent with a low-grade glioma. **(B)** Coronal T2-weighted (left) and post-contrast T1-weighted (right) MRI revealing a well-circumscribed, hyperintense mass in the left temporal lobe. The lesion exhibits sharp borders and associated cystic components, aligning with histologic features of a pilocytic astrocytoma. **(C)** Axial T2-weighted (left) and post-contrast T1-weighted (right) MRI showing the lesion’s lateral and anterior extent, including proximity to the hippocampus and temporal horn. No evidence of leptomeningeal dissemination is observed.

**Figure 2:**
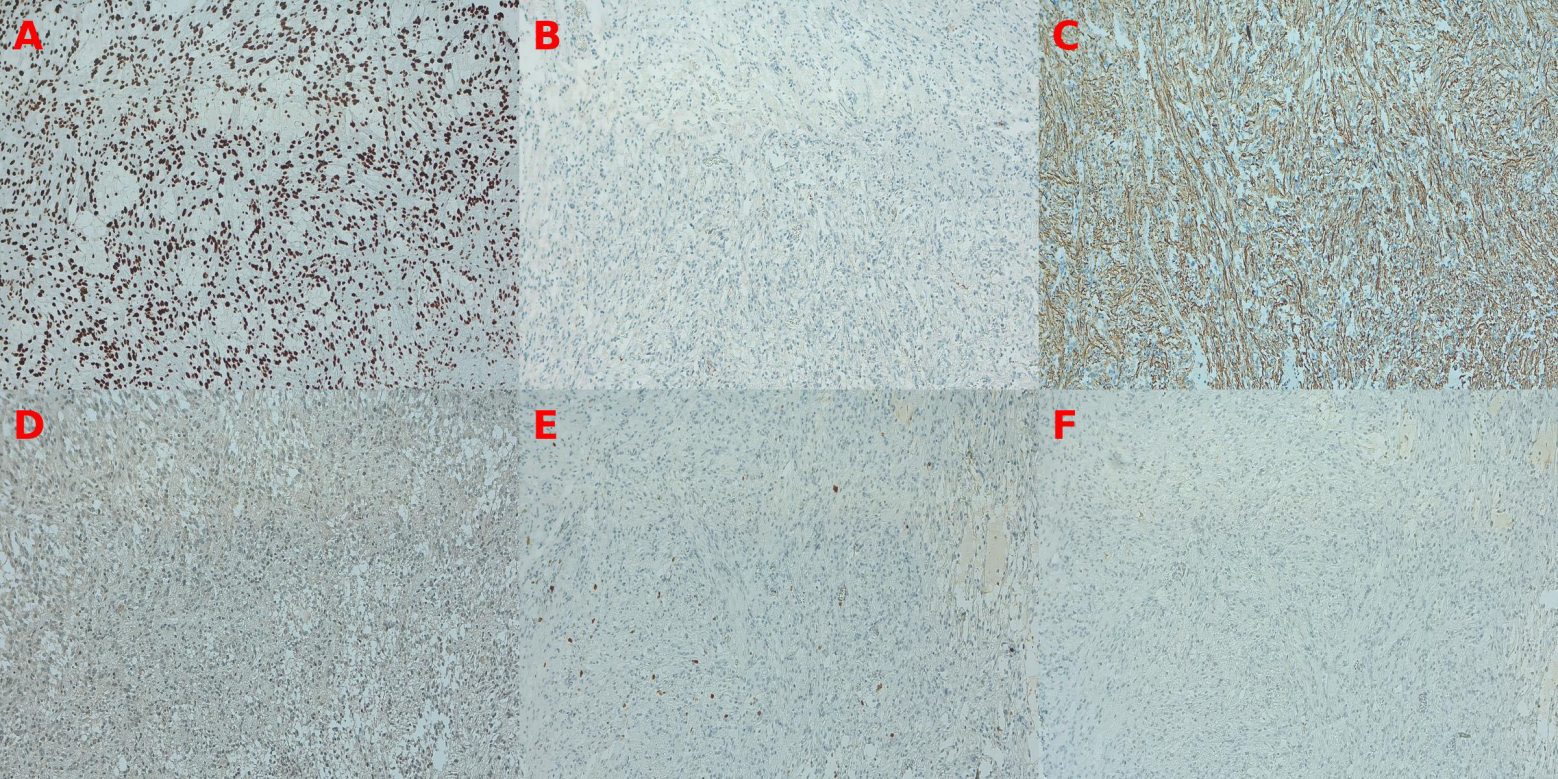
Immunohistochemical panel investigation (100x). **(A)** ATRX: Tumor cells exhibit retained nuclear ATRX expression. **(B)** EGFR: No membranous or cytoplasmic EGFR staining is observed. **(C)** GFAP: Strong cytoplasmic GFAP positivity highlights piloid processes and fibrillary astrocytic differentiation. **(D)** IDH1: No diffuse mutant-type immunoreactivity is present, with only occasional background staining. **(E)** Ki-67: Scattered nuclear labeling indicates a low proliferative index (~2–3%). **(F)** p53: Absence of aberrant nuclear accumulation, consistent with a negative p53 immunoprofile. Findings correspond to the immunohistochemical results described in the final surgical pathology report.

A 31-year-old female presented with new-onset epileptic seizures. MRI revealed a partially cystic, well-circumscribed lesion in the left temporal pole (refer to **Figures 1A-C**). The patient underwent a craniotomy and complete resection of her tumor, and tissue samples were analyzed using a combination of histopathology, immunohistochemistry, and next-generation sequencing (NGS). Histological examination showed a biphasic astrocytic tumor with piloid morphology and oligodendroglial-like features. Immunohistochemistry showed positive GFAP expression and negative CD34, EGFR, and p53. The Ki-67 index was low (2–4%), supporting a low-grade phenotype. (refer to **Figure 2**) As diagnostic interpretation, the final histology report state that microscopy evidence described is in keeping with Grade 1 Pilocytic Astrocytoma with cystic constituent. A postoperative scan demonstrated complete resection of the lesion. Consequently, no further adjuvant treatment was indicated, and the patient was enrolled in a routine imaging surveillance program.

### Methodology

For fusion detection, the Archer FUSIONPlex Pan Solid Tumor v2 panel was employed, enabling RNA-based detection of known and novel gene fusions, analyzed by ArcherDX. For DNA variant analysis, the Agilent SureSelect Cancer CGP assay was used to identify mutations and copy number variations, analyzed with Franklin by Genoox. Molecular testing detected an ASAP1exon29::BRAFexon9 fusion, breakpoint chr8:13107020-chr7:140487384. No other informative molecular markers were detected. Target specific primers were designed, and fusion was confirmed with amplicon NGS on cDNA.

For the bioinformatic investigation of the fusion, Deep Learning-Based Fusion Breakpoint Validation (FusionAI) was used to rigorously evaluate the candidate fusion and distinguish bona fide fusion events from potential RNA-sequencing artifacts (8–10). The ASAP1exon29::BRAFexon9 fusion detected by RNA sequencing was computationally validated using FusionAI, a deep learning–based fusion breakpoint verification tool that distinguishes true genomic rearrangements from RNA-sequencing artifacts by analyzing the underlying DNA sequence context. Using RNA-derived breakpoint coordinates, a 20 kb composite genomic sequence spanning ±5 kb of both fusion partners was generated, one-hot encoded, and analyzed using the pre-trained FusionAI convolutional neural network to predict fusion likelihood and sequence-level feature importance. Local sequence contributions were quantified using a sliding-window masking strategy, and high-impact regions were further annotated across 44 genomic feature categories using the FusionAI_genomic_features.R annotation workflow, encompassing repetitive elements, regulatory regions, chromatin states, and structural variation–associated features. Full details of model architecture, training data, negative control curation, and annotation procedures are provided in the **Supplementary Data Sheet 1**.

## Discussion

The most common fusion in pilocytic astrocytoma (PA), results from a tandem duplication at chromosome 7q34 that fuses the 5′ region of KIAA1549 with the 3′ kinase domain of BRAF. This event eliminates BRAF exons 1–8, which encode the RAS-binding and auto-inhibitory domains, and retains exons 9–18, encoding the active kinase region. The resulting chimeric protein lacks normal regulatory control, leading to constitutive activation of the MAPK/ERK signaling pathway independent of upstream RAS activation. This continuous signaling promotes glial cell proliferation and survival, driving the development of the low-grade, slow-growing phenotype typical of pilocytic astrocytoma. (11, 12)

The ASAP1 gene (also known as DDEF1 or AMAP1) is widely expressed across multiple human tissues, with particularly notable expression in endothelial cells, neural and glial cell types, as well as various epithelial and mesenchymal lineages. According to RNA expression data from the *Human Protein Atlas*, ASAP1 clusters within the “lymphoid tissue & bone marrow” and “brain” expression groups. Single-cell transcriptomic data further indicate high ASAP1 expression levels in oligodendrocyte precursor cells (≈583 nTPM), excitatory neurons (≈334 nTPM), astrocytes (≈196 nTPM), and other glial and neuronal cell types.

**Figure 3:**
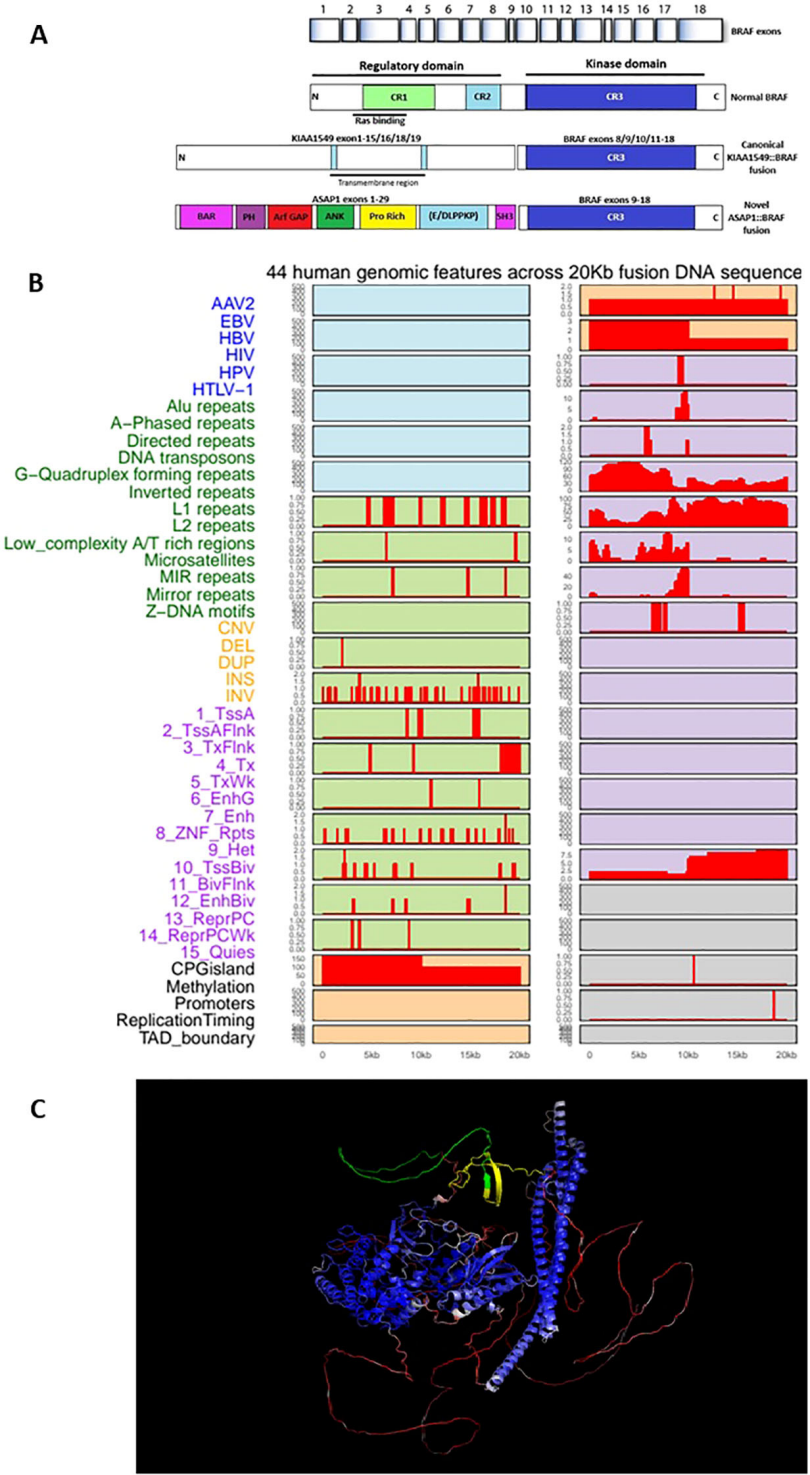
**(A)** Structural organization of the BRAF gene and its fusion variants. Normal BRAF contains an N-terminal regulatory domain (CR1–CR2) and a C-terminal kinase domain (CR3). The canonical KIAA1549::BRAF fusion retains the intact CR3 kinase domain (exons 8/9–18), resulting in constitutive activation. The novel ASAP1::BRAF fusion links ASAP1 exons 1–29—encoding BAR, PH, ArfGAP, ANK, proline-rich, and SH3 domains—to BRAF exons 9–18, preserving the CR3 kinase domain. Abbreviations: BAR, Bin-Amphiphysin-Rvs domain; PH, pleckstrin homology; ArfGAP, ADP-ribosylation factor GTPase-activating protein; ANK, ankyrin repeats; SH3, Src homology 3 domain. **(B)** Genomic landscape across the 20 kb region spanning the ASAP1::BRAF fusion. The plot displays 44 genomic annotations within a window constructed from the 5′ and 3′ partner flanking sequences, with the fusion breakpoint centered at 10 kb. Tracks are grouped by functional category: viral integration sites (blue), repetitive elements (green), structural variants (yellow), chromatin states (purple), and regulatory features (gray/orange). Red bars denote the presence and relative density of each feature along the sequence; blank regions indicate absence. **(C)** Structural context of the ASAP1::BRAF fusion interface. The predicted fusion protein is colored by stability (blue = stable core; red = flexible regions). The ASAP1-to-BRAF crossover point (yellow–green) occurs within a well-structured region rather than a disordered loop, highlighting that the fusion joins two stably folded elements.

The detected ASAP1::BRAF fusion, in which ASAP1 exon 29 is joined in-frame to BRAF exon 9, is very likely to behave biologically similar to the well-characterized KIAA1549::BRAF and other oncogenic BRAF fusions seen in low-grade gliomas. This is because the breakpoint within BRAF exon 9 preserves the entire C-terminal kinase domain (exons 9–18) while removing the N-terminal RAS-binding and auto-inhibitory regions (exons 1–8) that normally keep BRAF activity under tight control. The replacement of these regulatory domains with the N-terminal portion of ASAP1, which contains scaffolding and oligomerization motifs, can promote constitutive dimerization and activation of the BRAF kinase. As a result, the fusion protein is predicted to signal independently of upstream RAS activation, driving persistent MAPK/ERK pathway activation and cellular proliferation—mirroring the mechanism of other oncogenic BRAF fusions. Functionally, such fusions are insensitive to classical BRAF inhibitors but may respond to MEK inhibitors or RAF dimer inhibitors, reflecting their distinct dimer-driven signaling mechanism. (Refer to **Figure 3**) In this context, type II RAF inhibitors such as Tovorafenib and Belvarafenib are of particular interest in MAPK-driven brain tumors, as they bind the inactive (DFG-out) conformation of RAF kinases and effectively inhibit RAF dimers without causing paradoxical MAPK activation. Emerging clinical data suggest that these agents may offer a more rational targeted strategy for BRAF fusion positive gliomas and other MAPK-activated CNS tumors compared with first-generation BRAF inhibitors.

Deep learning–based evaluation and genomic feature analysis collectively substantiate the structural validity and mechanistic plausibility of the ASAP1::BRAF fusion. FusionAI assigned a fusion probability of 0.99999845 (error rate: 1.53 × 10^-6^), indicating that the local DNA sequence conforms closely to instability signatures characteristic of biologically authenticated fusion breakpoints. Although not a substitute for orthogonal molecular confirmation, this computational evidence strongly supports a bona fide genomic rearrangement rather than an RNA-sequencing artifact. Complementary mapping of 44 genomic features across the 20 kb interval centered on the breakpoint (refer to **Figure 3**) revealed pronounced enrichment of instability-associated elements—including Alu repeats and L1 retrotransposons—directly flanking the junction. This configuration closely parallels the instability architecture documented in canonical fusions like BCR::ABL1 and TMPRSS2::ERG, placing the ASAP1::BRAF rearrangement within the established framework of sequence-driven genomic fragility. Further bioinformatic interrogation indicates that the rearrangement most likely arose from endogenous structural instability rather than exogenous mutagenesis. The absence of signal across viral integration tracks effectively excludes oncoviral insertion as a contributing mechanism (13). In contrast, dense clustering of Alu and L1 elements at the breakpoint provides a mechanistic substrate for double-strand breaks and erroneous repair through non-allelic homologous recombination (14–16). Structural-variant annotations show that both partner loci reside within evolutionarily fragile, late-replicating regions that exhibit heightened susceptibility to breakage under replicative stress (17). Chromatin-state profiling further demonstrates that the breakpoint lies within euchromatin enriched for active promoter and enhancer signatures, a configuration known to expose DNA to endonucleases and facilitate rearrangements (18). Regulatory features—including CpG islands and high promoter activity—underscore the transcriptionally active nature of the locus, which increases torsional strain, fosters replication–transcription conflicts, and promotes R-loop formation, all contributing to transcription-associated mutagenesis (19; N. 20). These observations align with reports that BRAF fusions in melanoma frequently arise in contexts of heightened cellular and genomic stress (21). Taken together, the deep learning predictions and genomic landscape converge on a model in which intrinsic sequence composition, chromatin accessibility, and regulatory activity synergistically predisposed the ASAP1 and BRAF loci to double-strand breakage and rearrangement, giving rise to the observed fusion.

## Conclusion

This case illustrates the diagnostic and clinical significance of comprehensive molecular profiling in low-grade gliomas. The identification of a previously unreported ASAP1::BRAF fusion in a young adult pilocytic astrocytoma expands the growing gene list of BRAF fusion partners and reinforces the biological diversity underpinning MAPK-driven tumors. By preserving the intact BRAF kinase domain while removing the N-terminal regulatory elements, the fusion is predicted to behave similarly to established oncogenic BRAF rearrangements, thereby functioning as a driver of MAPK pathway activation.

Integrating RNA-level fusion detection with concordant deep-learning analyses markedly increased confidence that the rearrangement represents a true genomic event. With growing data showing that BRAF fusion–driven tumors may respond to MAPK-pathway inhibitors, accurate detection of these fusions is clinically meaningful and could shape treatment decisions in the event of disease recurrence.

Although the patient did not receive MAPK inhibitors as first-line therapy due to complete surgical resection, the presence of a BRAF fusion has been documented in her clinical record. Targeted MAPK inhibition has been identified as a therapeutic option in the event of tumor recurrence or relapse.

Overall, this finding broadens the molecular landscape of BRAF-altered gliomas and underscores the need for continued investigation of rare fusion events, both to refine diagnostic classification and to guide precision-based management strategies for affected patients.

## Data Availability

The original contributions presented in the study are included in the article/**Supplementary Material**, further inquiries can be directed to the corresponding author/s.

## References

[1] Penman CL Faulkner C Lowis SP Kurian KM . Current understanding of BRAF alterations in diagnosis, prognosis and therapeutic targeting in paediatric low grade gliomas. Front Oncol. (2015) 5:54. doi: 10.3389/fonc.2015.00054, PMID: 25785246 PMC4347423

[2] Ryall S Zapotocky M Fukuoka K Nobre L Guerreiro Stucklin A Bennett J . Integrated molecular and clinical analysis of 1,000 pediatric low-Grade gliomas. Cancer Cell. (2020) 37. doi: 10.1016/j.ccell.2020.03.011, PMID: 32289278 PMC7169997

[3] Schreck KC Langat P Bhave VM Li T Woodward E Pratilas CA . Integrated molecular and clinical analysis of BRAF-mutant glioma in adults. NPJ Precis Oncol. (2023) 7. doi: 10.1038/s41698-023-00359-y, PMID: 36854806 PMC9975216

[4] Lind KT Chatwin HV Desisto J Coleman P Sanford B Donson AM . Novel RAF fusions in pediatric low-grade gliomas demonstrate MAPK pathway activation. J Neuropathology Exp Neurol. (2021) 80. doi: 10.1093/jnen/nlab110, PMID: 34850053

[5] Ali RH Almanabri M Ali NY Alsaber AR Khalifa NM Hussein R . Clinicopathological analysis of BRAF and non-BRAF MAPK pathway-altered gliomas in paediatric and adult patients: a single-institution study of 40 patients. J Clin Pathol. (2024). doi: 10.1136/jcp-2023-209318, PMID: 38195220 PMC11874301

[6] Houghton PJ . Advances in the treatment of BRAF-mutant low-grade glioma with MAPK inhibitors. Trans Pediatr. (2024) 13. doi: 10.21037/tp-23-541, PMID: 38590382 PMC10998999

[7] Crotty EE Sato AA Abdelbaki MS . Integrating MAPK pathway inhibition into standard-of-care therapy for pediatric low-grade glioma. Front Oncol. (2025) 15:1520316. doi: 10.3389/fonc.2025.1520316, PMID: 40007996 PMC11850343

[8] Kim P Tan H Liu J Yang M Zhou X . FusionAI: Predicting fusion breakpoint from DNA sequence with deep learning. IScience. (2021) 24. doi: 10.1016/j.isci.2021.103164, PMID: 34646994 PMC8501764

[9] Kim P Tan H Liu J Kumar H Zhou X . FusionAI, a DNA-sequence-based deep learning protocol reduces the false positives of human fusion gene prediction. STAR Protoc. (2022) 3. doi: 10.1016/j.xpro.2022.101185, PMID: 35252882 PMC8892011

[10] Kim P Tan H Liu J Lee H Jung H Kumar H . FusionGDB 2.0: Fusion gene annotation updates aided by deep learning. Nucleic Acids Res. (2022) 50. doi: 10.1093/nar/gkab1056, PMID: 34755868 PMC8728198

[11] Jones DTW Kocialkowski S Liu L Pearson DM Bäcklund LM Ichimura K . Tandem duplication producing a novel oncogenic BRAF fusion gene defines the majority of pilocytic astrocytomas. Cancer Res. (2008) 68. doi: 10.1158/0008-5472.CAN-08-2097, PMID: 18974108 PMC2577184

[12] Pfister S Janzarik WG Remke M Ernst A Werft W Becker N . BRAF gene duplication constitutes a mechanism of MAPK pathway activation in low-grade astrocytomas. J Clin Invest. (2008) 118. doi: 10.1172/JCI33656, PMID: 18398503 PMC2289793

[13] Akagi K Li J Broutian TR Padilla-Nash H Xiao W Jiang B . Genome-wide analysis of HPV integration in human cancers reveals recurrent, focal genomic instability. Genome Res. (2014) 24. doi: 10.1101/gr.164806.113, PMID: 24201445 PMC3912410

[14] Mendez-Dorantes C Burns KH . LINE-1 retrotransposition and its deregulation in cancers: implications for therapeutic opportunities. Genes Dev. (2023) 37. doi: 10.1101/gad.351051.123, PMID: 38092519 PMC10760644

[15] Roukos V Misteli T . The biogenesis of chromosome translocations. Nat Cell Biol. (2014) 16. doi: 10.1038/ncb2941, PMID: 24691255 PMC6337718

[16] Song X Beck CR Du R Campbell IM Coban-Akdemir Z Gu S . Predicting human genes susceptible to genomic instability associated with Alu/Alu-mediated rearrangements. Genome Res. (2018) 28. doi: 10.1101/gr.229401.117, PMID: 29907612 PMC6071635

[17] Glover TW Arlt MF Casper AM Durkin SG . Mechanisms of common fragile site instability. Hum Mol Genet. (2005) 14. doi: 10.1093/hmg/ddi265, PMID: 16244318

[18] Mansisidor AR Risca VI . Chromatin accessibility: methods, mechanisms, and biological insights. Nucleus. (2022) 13. doi: 10.1080/19491034.2022.2143106, PMID: 36404679 PMC9683059

[19] Crossley MP Bocek M Cimprich KA . R-loops as cellular regulators and genomic threats. Mol Cell. (2019) 73. doi: 10.1016/j.molcel.2019.01.024, PMID: 30735654 PMC6402819

[20] Kim N Jinks-Robertson S . Transcription as a source of genome instability. Nat Rev Genet. (2012) 13. doi: 10.1038/nrg3152, PMID: 22330764 PMC3376450

[21] Botton T Talevich E Mishra VK Zhang T Shain AH Berquet C . Genetic heterogeneity of BRAF fusion kinases in melanoma affects drug responses. Cell Rep. (2019) 29. doi: 10.1016/j.celrep.2019.09.009, PMID: 31618628 PMC6939448

