# Supplementary material for: Case Report: Novel ASAP1::BRAF fusion in a young adult with low-grade temporal lobe glioma
Source: Front Oncol. 2026 Jan 23;16:1763047. doi: 10.3389/fonc.2026.1763047 (PMC12878647; doi:10.3389/fonc.2026.1763047)
Supplement: Supplementary file 1 [file DataSheet1.docx]

**Supplementary**

For the bioinformatic investigation of the fusion, Deep Learning-Based Fusion Breakpoint Validation (FusionAI) was used to rigorously evaluate the candidate fusion and distinguish bona fide fusion events from potential RNA-sequencing artifacts. Although the fusion was initially detected at the RNA level, FusionAI performs validation by analyzing the underlying genomic DNA context. The model is designed around the premise that authentic RNA fusions arise from specific genomic instabilities—such as repetitive elements or characteristic sequence motifs—embedded within the DNA. Using the coordinates of the RNA-derived exon junctions, FusionAI extracts and interrogates the latent “breakage code” within the primary DNA sequence that may have facilitated the chromosomal rearrangement.

**Model Architecture and Training**

FusionAI is implemented as a convolutional neural network (CNN) comprising two convolutional layers with filter dimensions of (20, 4) and (200, 1), followed by max-pooling, flattening, and two fully connected dense layers. The architecture contains approximately 2.67 million parameters. Training was performed on a balanced dataset that included ~26,000 fusion-positive exon junction breakpoints derived from TCGA RNA-seq data and ~26,000 fusion-negative controls. To ensure high specificity and minimize alignment-related artifacts, the negative set was stringently filtered to exclude paralogous genes, pseudogenes (using the Duplicated Genes and HUGO databases), and genomic regions annotated as repetitive by RepeatMasker.

**Input Processing and Feature Analysis**

For analysis of the candidate fusion, a 20 kb genomic sequence was constructed by concatenating the ±5 kb flanking regions from the breakpoints of both the 5′ and 3′ partner genes. This sequence was one-hot encoded and input into the pre-trained FusionAI model. After fusion-probability prediction, Feature Importance Scores (FIS) were calculated using a sliding-window masking approach (20 bp window, stride of 1) to quantify the contribution of local sequence motifs to the predicted fusion likelihood. To further contextualize these high-FIS regions, 44 genomic features—spanning viral integration sites, repetitive elements, structural variants, chromatin states, and regulatory annotations—were mapped using the FusionAI_genomic_features.R workflow.
